# Role of hematological biomarkers in predicting oncological outcomes of definitive chemoradiation in locally advanced vulvar carcinoma

**DOI:** 10.14440/jbm.2025.0104

**Published:** 2024-12-18

**Authors:** Maysa Al Hussaini, Ramiz Abuhijlih, Issa Mohamad, Abdallah Al-Ani, Fawzi Abuhijla

**Affiliations:** 1 Department of Pathology, King Hussein Cancer Center, Amman, 11941, Jordan; 2 Department of Radiation Oncology, King Hussein Cancer Center, Amman, 11941, Jordan; 3 Department of Scientific Affairs and Research, King Hussein Cancer Center, Amman, 11941, Jordan

**Keywords:** Vulvar cancer, Chemoradiation, Neutrophil–to–lymphocyte ratio, Platelet–to–lymphocyte ratio, Complete response

## Abstract

**Background:**

The systemic inflammatory response triggered by the carcinogenic process induces significant changes in a wide range of hematological biomarkers, impacting their levels, functions, and overall roles in the body’s physiological and pathological processes.

**Objective:**

To evaluate the value of pre-treatment hematological parameters in the prediction of clinical and radiological responses of locally advanced vulvar cancer to definitive chemoradiation.

**Methods:**

We retrospectively reviewed the medical records of patients treated at the King Hussein Cancer Center receiving definitive chemoradiation for pathologically confirmed locally advanced vulvar carcinoma. Response of the primary disease to treatment was classified as complete response (CR) if there was no clinically- or radiologically-confirmed residual disease at 12 weeks after completion of chemoradiation. Univariate analyses on complete response, progression-free survival (PFS), and overall survival (OS) were performed using clinical factors and pre-treatment hematological parameters.

**Results:**

A total of 30 patients were included, with a median age of 57.5 years and a median follow-up of 21 months. Of these, 24 patients (80%) achieved CR. Disease progression occurred in 11 patients (36.7%) during the follow-up period, and 9 (30%) died. Kaplan–Meier analysis demonstrated that only the neutrophil–to–lymphocyte ratio (NLR) (*p* = 0.007) and basophil–to–lymphocyte ratio (BLR) (*p* = 0.05) were predictive of OS. Conversely, PFS was significantly associated with white blood cell count (*p* = 0.042) and BLR (*p* = 0.004). Receiver operating characteristic (ROC) analysis indicated that NLR and BLR had significant predictive power for survival at the following cutoffs: 3.4 and 0.035, respectively. When categorized by ROC values, BLR was significantly associated with response to treatment (*p* = 0.026). Moreover, both NLR and BLR were significantly associated with OS and PFS.

**Conclusion:**

Pre-treatment NLR and BLR may be useful predictive markers for clinical and radiological response, as well as for oncological outcomes in locally advanced vulvar cancer treated with definitive chemoradiation.

## 1. Introduction

Vulvar cancer is the fourth most common gynecological malignancy, with squamous cell carcinoma accounting for 95% of all cases.^1^ The management of non-metastatic vulvar cancer is primarily dictated by the tumor stage. For early-stage disease, surgery is usually indicated, followed by observation, radiation therapy, or chemoradiation, depending on histopathological findings.^2^,^3^ In contrast, definitive chemoradiation is considered the most appropriate treatment for locally advanced lesions.^4^

The pathogenesis of vulvar cancer has been linked to various inflammatory processes, including human papillomavirus (HPV) infections and autoimmune conditions such as psoriasis.^5^ Moreover, inflammatory proteins have been implicated in the progression of vulvar carcinogenesis.^6^ Bartl *et al*.^7^ demonstrated that the systemic immune-inflammation index is an independent prognostic factor for survival in patients with invasive vulvar carcinoma.

The systematic inflammatory response to neoplastic disease is characterized by elevated levels of proinflammatory cytokines, leukocyte migration, and increased platelet counts. Several markers, including C-reactive protein (CRP), albumin level, blood cell count, and absolute blood cell ratios, can reflect this inflammatory process.^8^ These markers, often derived from complete blood counts, have shown promising utility in the prognostication of both gynecological and non-gynecological malignancies. However, their predictive value in vulvar cancer remains under-explored due to a limited number of studies. Therefore, this study aimed to evaluate the predictive power of hematological biomarkers for clinical and radiological responses, as well as oncological outcomes, in vulvar cancer.

## 2. Materials and methods

### 2.1. Materials

We conducted a retrospective chart review of patients diagnosed with locally advanced vulvar carcinoma (based on the International Federation of Gynecology and Obstetrics [FIGO] staging), who were treated with definitive chemoradiation. All included cases were confirmed through biopsy, either performed at the King Hussein Cancer Center (KHCC) or re-evaluated by an expert gynecological oncology pathologist (M.A.H.) in cases whose biopsies were conducted externally. Patients who did not complete their radical radiation course did not receive chemotherapy, or underwent neoadjuvant radiation were excluded. The following variables were reviewed from patients’ charts: age at diagnosis (in years), stage at presentation, pre-treatment hematological markers (white blood cell count [WBC], absolute neutrophil count [ANC], absolute lymphocyte count [ALC], absolute eosinophile count [AEC], absolute monocyte count [AMC], absolute basophile count [ABC], platelet count [Pc], hemoglobin level [Hb], the neutrophil–to–lymphocyte ratio [NLR], platelet–to–lymphocyte ratio [PLR], monocyte–to–lymphocyte ratio [MLR], eosinophil–to–lymphocyte ratio [ELR], basophil–to–lymphocyte ratio [BLR]), and survival markers (overall survival [OS] and progression-free survival [PFS]). The study protocol was approved by the KHCC Institutional Review Board (Approval #20KHCC117).

### 2.2. Methods

Radiation therapy was delivered at a dose ranging 63 – 70 Gray (Gy) over 33 – 35 fractions (fx), with each fx delivering 1.8 – 2 Gy. Simulation and treatment were performed in a supine position using either frog-leg or straight-leg technique. Volumetric arc radiation therapy with cone beam computed tomography (CT) image guidance was utilized.

Chemotherapy was administered as a radiosensitizer during radiation therapy, using weekly intravenous cisplatin (dose: 40 mg/m^2^). Throughout the chemoradiation course, patients were monitored for complete blood counts and clinically assessed on a weekly basis. All patients in this cohort tolerated the treatment without discontinuation.

Following the completion of definitive chemoradiation, patients were followed up at the 2-week mark to assess treatment toxicity. At the 3-month follow-up, patients were examined for treatment response through clinical evaluation (physical examination) and pelvic radiological assessment (magnetic resonance imaging [MRI]). MRI scans have been proven to be an effective tool for evaluating loco-regional disease in vulvar cancer and post-therapeutic response. Pre- and post-chemoradiation MRI scan findings were compared to measure tumor response. Complete response (CR) was defined as the absence of clinical or radiological residual disease 12 weeks after completion of the chemoradiation course.

### 2.3. Statistical analysis

All analyses were performed using R software (version 4.0.2, R Core Team, Austria, 2020). Descriptive statistics were utilized to summarize data. Categorical variables were presented as frequencies with associated percentages, whereas continuous variables were expressed as medians with interquartile ranges. Hematological markers (i.e., NLR, PLR, and Hb) were categorized according to their median values. Associations between categorized hematological markers and response to therapy (CR versus disease progression [DP]) were assessed using Fischer’s exact test. Differences in hematological markers between different groups were examined using the Mann–Whitney U test and presented as Gardner-Altman plots. Spearman’s rank correlation was used to investigate correlations between hematological markers. Receiver operating curve (ROC) analysis was conducted for all markers. Survival curves for OS and PFS were generated using the Kaplan–Meier method and compared using the log-rank test. Survival parameters were given as means with 95% confidence intervals. A *p* ≤ 0.05 was considered statistically significant for all tests.

## 3. Results

### 3.1. Characteristics of included cohort

A total of 30 patients were included in the final analysis, and their characteristics are summarized in Table 1. The mean age at diagnosis for the entire sample was 57.5 years (range: 46.7 – 69.0 years). The majority of tumors were located in the central/clitoral area (*n* = 13; 43%), and the majority of the patients were classified as FIGO stage III (*n* = 21; 70%) and FIGO grade II (*n* = 23; 77%). The median follow-up duration lasted for 25.1 months, with a mean time of 31.4 months. A total of 24 patients (80%) achieved CR by 12 weeks post-treatment. DP took place in 11 patients (36.7%) during the follow-up period, and death occurred in 9 patients (30%).

**Table 1:** Characteristics of included patients

Variables	*n* (%)
Age (years)	
30 – 39	8 (27)
40 – 49	8 (27)
50 – 59	7 (23)
60 – 69	5 (17)
70 – 79	1 (3)
80 – 89	1 (3)
Site	
Clitoral	13 (43)
Labia Minora	9 (30)
Labia Majora	1 (3)
Periurethral	7 (23)
FIGO Stage	
II	4 (13)
III	21 (70)
IV	5 (17)
Radiation dose	
63 – 65 Gy	17 (57)
>65 Gy	13 (43)
Tumor grade	
1	2 (6)
2	23 (77)
3	5 (17)
p16	
Positive	6 (20)
Negative	18 (60)
NA	6 (20)
p53	
Mutated	17 (57)
Negative	6 (20)
NA	7 (23)

Abbreviations: FIGO: The International Federation of Gynecology and Obstetrics; p16: Cyclin-dependent kinase inhibitor 2A; p53: Cellular tumor antigen p53.

### 3.2. Hematological markers

Table 2 lists the hematological characteristics of the included participants. Of the hematological markers examined, only ANC (*p* = 0.017) and WBC (*p* = 0.017) were significantly associated with CR at 12 weeks post-treatment. Lymphocyte-based ratios, such as NLR (*p* = 0.169), PLR (*p* = 0.999), MLR (*p* = 0.651), and ELR (*p* = 0.651), were not significantly associated with treatment response, as shown in Table 3.

**Table 2:** Hematological characteristics of the entire cohort

Variable	Median	IQR	Min	Max	Mean	SD
Hb	11.75	10.32 – 12.90	7.10	14.50	11.64	1.80
Plt	287.00	244.50 – 378.25	143.00	664.00	320.43	118.97
ANC	4.95	3.93 – 6.67	2.08	15.82	5.69	2.84
ALC	1.68	1.28 – 2.20	0.77	3.36	1.79	0.63
AEC	0.10	0.05 – 0.18	0.00	0.37	0.12	0.09
AMC	0.59	0.45 – 0.67	0.10	1.05	0.58	0.19
ABC	0.05	0.03 – 0.06	0.02	0.13	0.05	0.03
WBC	8.05	6.52 – 9.22	4.23	17.40	8.21	2.8
NLR (ANC/ALC)	2.68	1.95 – 4.42	0.89	15.41	3.81	3.10
PLR (Plt/ALC)	189.45	111.73 – 274.72	62.07	642.86	206.41	119.97
MLR (AMC/ALC)	0.31	0.25 – 0.44	0.08	0.88	0.36	0.19
ELR (AEC/ALC)	0.04	0.03 – 0.10	0.00	0.36	0.08	0.07
ABCr (ABC/ALC)	0.28	0.02 – 0.05	0.01	0.07	0.33	0.02

Abbreviations: ABC: Absolute basophil count; ABCr (ABC/ALC): Absolute basophil count ratio; AEC: Absolute eosinophil count; ALC: Absolute lymphocyte count; AMC: Absolute monocyte count; ANC: Absolute neutrophil count; ELR (AEC/ALC): Eosinophil–to–lymphocyte ratio; Hb: Hemoglobin level; IQR: Interquartile range; Max: Maximum; Min: Minimum; MLR (AMC/ALC): Monocyte–to–lymphocyte ratio; NLR (ANC/ALC): Neutrophil–to–lymphocyte ratio; PLR (Plt/ALC): Platelet–to–lymphocyte ratio; Plt: Platelet count; SD: Standard deviation; WBC: White blood cell count.

**Table 3:** Associations between hematological markers and response to treatment

Variable	Value	CR (%)	DP (%)	*p*-value	*OR*
Hb	Below median	10 (41.7)	5 (83.3)	0.169	0.143 ( 0.014 – 1.418)
	Above median	14 (58.3)	1 (16.7)		
Plt	Below median	12 (50.0)	3 (50.0)	0.999	1.000 (0.167 – 5.985)
	Above median	12 (50.0)	3 (50.0)		
ANC	Below median	15 (62.5)	0 (0.0)	0.017*	–
	Above median	9 (37.5)	6 (100.0)		
ALC	Below median	10 (41.7)	5 (83.3)	0.169	0.143 ( 0.014 – 1.418)
	Above median	14 (58.3)	1 (16.7)		
AEC	Below median	13 (54.2)	2 (33.3)	0.651	2.364 (0.361 – 15.455)
	Above median	11 (45.8)	4 (66.7)		
AMC	Below median	13 (54.2)	2 (33.3)	0.651	2.364 (0.361 – 15.455)
	Above median	11 (45.8)	4 (66.7)		
ABC	Below median	12 (50.0)	3 (50.0)	0.999	1.000 (0.167 – 5.985)
	Above median	12 (50.0)	3 (50.0)		
WBC	Below median	15 (62.5)	0 (0.0)	0.017*	–
	Above median	9 (37.5)	6 (100.0)		
NLR	Below median	14 (58.3)	1 (16.7)	0.169	7.000 (0.705 – 69.490)
	Above median	10 (41.7)	5 (83.3)		
PLR	Below median	12 (50.0)	3 (50.0)	0.999	1.000 (0.167 – 5.985)
	Above median	12 (50.0)	3 (50.0)		
MLR	Below median	13 (54.2)	2 (33.3)	0.651	2.364 (0.361 – 15.455)
	Above median	11 (45.8)	4 (66.7)		
ELR	Below median	13 (54.2)	2 (33.3)	0.651	2.364 (0.361 – 15.455)
	Above median	11 (45.8)	4 (66.7)		
ABCr	Below median	14 (58.3)	1 (16.7)	0.169	7.000 (0.705 – 69.490)
	Above median	10 (41.7)	5 (83.3)		

Notes: All associations were calculated using Fisher’s exact test.

* Indicates statistical significance of (*p*≤0.05). CR and DP are expressed in the number of patients (percentage).

Abbreviations: ABC: Absolute Basophil Count; ABCr: Absolute basophil count ratio; AEC: Absolute eosinophil count; ALC: Absolute lymphocyte count;

AMC: Absolute monocyte count; ANC: Absolute neutrophil count; CR: Complete response; DP: Disease progression; ELR: Eosinophil–to–lymphocyte ratio;

Hb: Hemoglobin level; MLR: Monocyte–to–lymphocyte ratio; NLR: Neutrophil–to–lymphocyte ratio; *OR*: Odds ratio; PLR: Platelet–to–lymphocyte ratio; Plt: Platelet count; WBC: White blood cell count.

**Figure S1:**
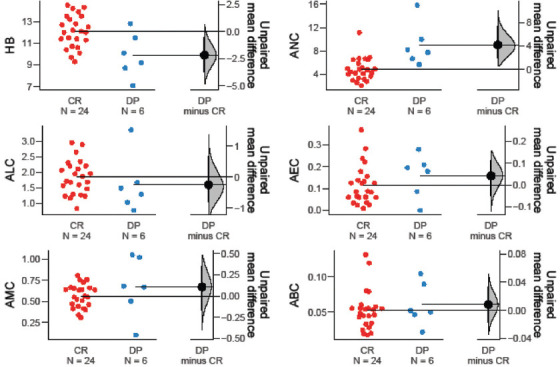
Unpaired mean differences between DP and CR in HB level, ALC, AMC, ANC, AEC, and ABC Abbreviations: DP: Disease progression; CR; Complete response; HB: Hemoglobin; ALC: Absolute lymphocyte count, AMC: Absolute monocyte count; ANC: Absolute neutrophil count; AEC: Absolute eosinophil count; ABC: Absolute basophil count.

**Figure S2:**
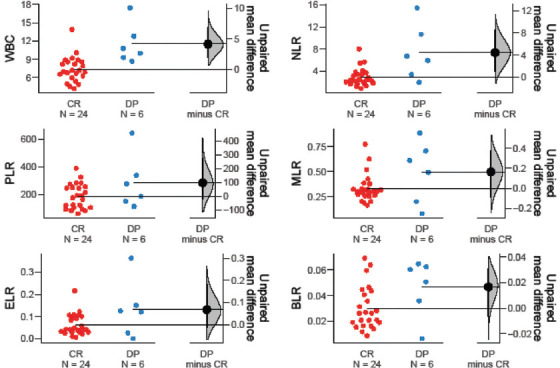
Unpaired mean differences between DP and CR in WBC count, PLR, ELR, NLR, MLR, and BLR Abbreviations: DP: Disease progression; CP: Complete response; WBC: White blood cell; PLR: Platelet–to–lymphocyte ratio; ELR: Eosinophil–to–lymphocyte ratio; NLR: Neutrophil–to–lymphocyte ratio; MLR: Monocyte–to–lymphocyte ratio; BLR: Basophil–to–lymphocyte ratio.

**Figure S3:**
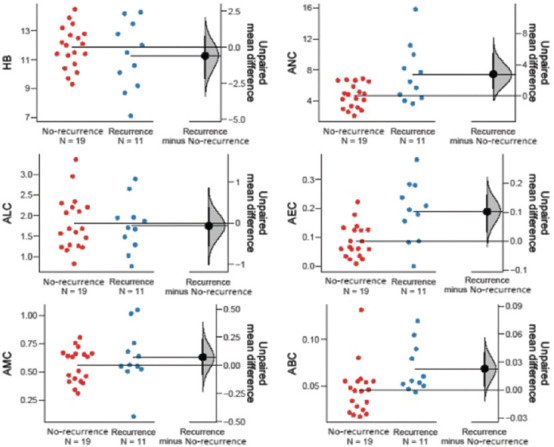
Unpaired mean differences between recurrence and no recurrence in HB level, ALC, AMC, ANC, AEC, and ABC Abbreviations: HB: Hemoglobin; ALC: Absolute lymphocyte count; AMC: Absolute monocyte count; ANC: Absolute neutrophil count; AEC: Absolute eosinophil count; ABC: Absolute basophil count.

**Figure S4:**
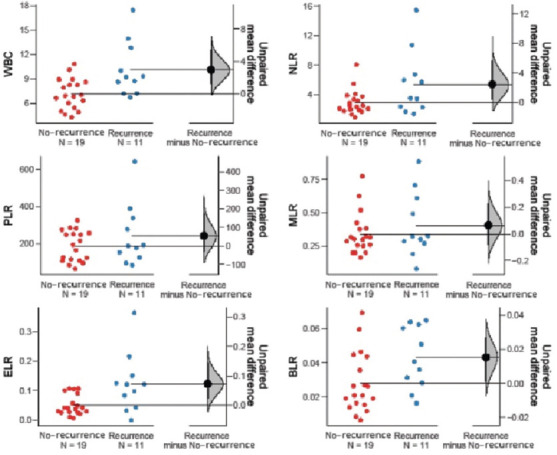
Unpaired mean differences between recurrence and no recurrence in WBC count, PLR, ELR, NLR, MLR, and BLR Abbreviations: WBC: Blood cell count; PLR: Platelet–to–lymphocyte ratio; ELR: Eosinophil–to–lymphocyte ratio; NLR: Neutrophil–to–lymphocyte ratio; MLR: Monocyte–to–lymphocyte ratio; BLR: Basophil–to–lymphocyte ratio.

**Figure S5:**
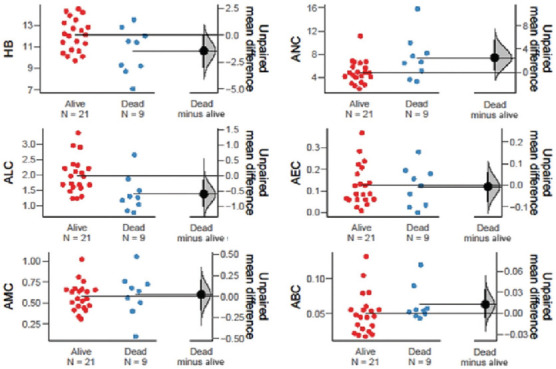
Unpaired mean differences between dead and alive cases in HB level, ALC, AMC, ANC, AEC, and ABC Abbreviations: HB: Hemoglobin; ALC: Absolute lymphocyte count; AMC: Absolute monocyte count; ANC: Absolute neutrophil count, AEC: Absolute eosinophil count; ABC: Absolute basophil count.

**Figure S6:**
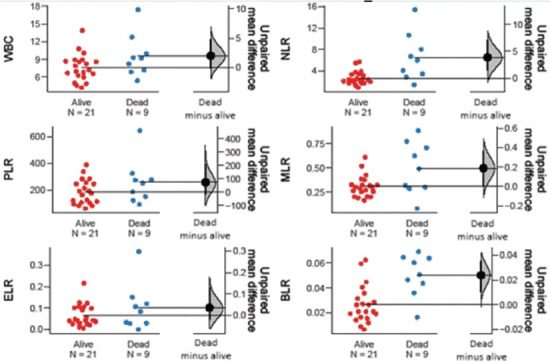
Unpaired mean differences between dead and alive cases in WBC count, PLR, ELR, NLR, MLR, and BLR Abbreviations: WBC: White blood cell; PLR: Platelet–to–lymphocyte ratio; ELR: Eosinophil–to–lymphocyte ratio; NLR: Neutrophil–to–lymphocyte ratio; MLR: Monocyte–to–lymphocyte ratio; BLR: Basophil–to–lymphocyte ratio.

Among the hematological markers, ANC (*p* = 0.001), WBC (*p* = 0.001), and NLR (*p* = 0.015) were significantly lower in treatment responders, whereas Hb (*p* = 0.021) was significantly higher in the same group. Moreover, ANC (*p* = 0.042), AEC (*p* = 0.005), ABC (*p* = 0.018), WBC (*p* = 0.005), ELR (*p* = 0.011), and BLR (*p* = 0.023) were significantly elevated in patients with recurrent disease. In patients who were pronounced dead at the time of data collection, markers such as ANC (*p* = 0.050), NLR (*p* = 0.004), and BLR (*p* = 0.002) were significantly higher than in those who were alive (Table 4 and Figures S1–S6).

**Table 4:** Mean survival time and differences among the included participants

Variable	Value	OS				PFS			
	
		Mean	Lower	Upper	*p*-value	Mean	Lower	Upper	*p*-value
Hb	Below median	45.6	26.6	64.7	0.196	44.5	24.1	64.9	0.544
	Above median	58.9	46.4	71.5		49.2	33.6	64.8	
Plt	Below median	43.5	27.1	59.9	0.267	43.5	26.8	60.2	0.741
	Above median	63.5	48.2	78.7		53.3	35.4	71.2	
ANC	Below median	68.8	56.1	81.5	0.032*	58.2	41.5	75.0	0.139
	Above median	32.6	16.6	48.6		33.9	17.4	50.5	
ALC	Below median	43.6	25.7	61.5	0.028*	52.5	33.7	71.3	0.803
	Above median	59.5	44.7	74.2		45.8	30.5	61.2	
AEC	Below median	39.3	28.9	49.7	0.887	43.3	34.1	52.5	0.172
	Above median	53.5	35.6	71.3		40.6	23.2	58.0	
AMC	Below median	57.4	40.2	74.5	0.525	48.0	29.7	66.2	0.969
	Above median	45.9	32.0	59.8		45.2	30.4	59.9	
ABC	Below median	63.8	48.9	78.7	0.292	64.2	49.5	78.9	0.098
	Above median	39.5	24.2	54.9		32.2	18.0	46.4	
WBC	Below median	63.2	47.9	78.5	0.142	63.7	48.5	78.8	0.042*
	Above median	35.3	17.6	52.9		30.4	15.3	45.5	
NLR	Below median	73.2	62.9	83.5	0.007*	58.2	41.5	75.0	0.139
	Above median	33.5	19.2	47.7		33.9	17.4	50.5	
PLR	Below median	52.4	36.9	67.9	0.487	45.2	29.4	60.9	0.934
	Above median	52.3	34.3	70.4		52.4	33.7	71.1	
MLR	Below median	55.9	40.7	71.2	0.221	48.1	33.2	63.9	0.472
	Above median	49.6	32.1	67.2		48.6	30.1	67.2	
ELR	Below median	40.4	30.7	50.1	0.718	43.5	34.5	52.5	0.109
	Above median	52.5	34.4	70.7		38.0	20.3	55.7	
ABCr	Below median	67.0	58.6	75.4	0.005*	62.3	50.6	74.0	0.004*
	Above median	37.1	18.9	55.2		30.4	12.5	48.3	

Note:

* Indicates statistical significance of (*p*<0.05).

Abbreviations: ABC: Absolute Basophil Count; ABCr: Absolute basophil count ratio, AEC: Absolute eosinophil count; ALC: Absolute lymphocyte count;

AMC: Absolute monocyte count; ANC: Absolute neutrophil count; ELR: Eosinophil–to–lymphocyte ratio; Hb: Hemoglobin level; MLR: Monocyte–to–lymphocyte ratio; NLR: Neutrophil–to–lymphocyte ratio; OS: Overall survival; PFS: Progression-free survival; PLR: Platelet–to–lymphocyte ratio; Plt: Platelet count; WBC: White blood cell count.

**Figure 1:**
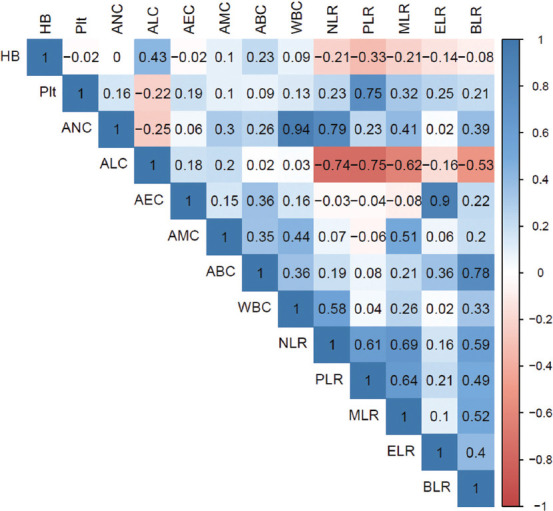
Correlations between different hematological markers Abbreviations: ABC: Absolute basophil count; AEC: Absolute eosinophil count; ALC: Absolute lymphocyte count; AMC: Absolute monocyte count; ANC: Absolute neutrophil count; ELR (AEC/ALC): Eosinophil–to–lymphocyte ratio; Hb: Hemoglobin level; MLR (AMC/ALC): Monocyte–to–lymphocyte ratio; NLR (ANC/ALC): Neutrophil–to–lymphocyte ratio; PLR (Plt/ALC): Platelet–to–lymphocyte ratio; Plt: Platelet count; WBC: White blood cell count; BLR: Basophil–to–lymphocyte ratio.

Figure 1 illustrates the correlations between different hematological markers. Notably, lymphocyte-based ratios, including NLR, PLR, MLR, and BLR, were all positively and significantly correlated with one another. However, ELR was only correlated with BLR. In addition to NLR, ANC was significantly correlated with WBC, MLR, and BLR.

### 3.3. Oncological outcomes

#### 3.3.1. Survival analysis

**Figure 2:**
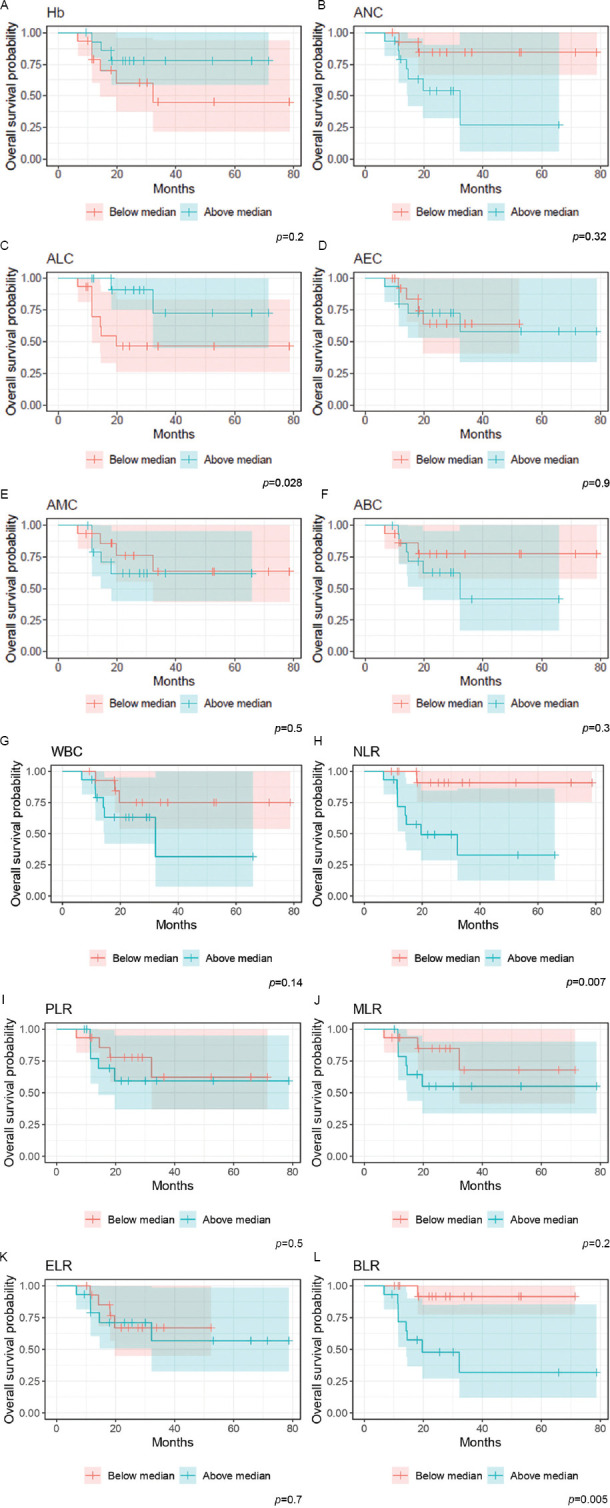
Overall survival Kaplan–Meier plots for overall survival based on A: Hb level, B: ANC, C: ALC, D: AEC, E: AMC, F: ABC, G: WBC, H: NLR. I: PLR, J: MLR, K: ELR and L: BLR hematological markers. Abbreviations: ABC: Absolute basophil count; AEC: Absolute eosinophil count; ALC: Absolute lymphocyte count; AMC: Absolute monocyte count; ANC: Absolute neutrophil count; BLR: Basophil–to–lymphocyte ratio; ELR: Eosinophil– to–lymphocyte ratio; Hb: Hemoglobin level; MLR: Monocyte–to–lymphocyte ratio; NLR: Neutrophil–to–lymphocyte ratio; PLR: Platelet–to–lymphocyte ratio; WBC: White blood cell count.

Kaplan–Meier analysis demonstrated that age (*p* = 0.035), ANC (*p* = 0.032), and ALC (*p* = 0.028) were significantly associated with OS. Of the ratios, only NLR (*p* = 0.007) and BLR (*p* = 0.05) were significantly associated with OS. On the other hand, PFS was significantly associated only with WBC (*p* = 0.042) and BLR (*p* = 0.004). Figure 2 display the Kaplan–Meier plots for the selected hematological markers.

#### 3.3.2. Receiver operating characteristic (ROC) analysis

ROC analysis indicated that NLR and BLR had significant predictive power for survival at the following cutoffs: 3.4 for NLR and 0.035 for BLR (Table S1). When categorized based on ROC values, BLR was significantly associated with response to treatment (*p* = 0.026), whereas NLR was not (*p* = 0.156) (Table S2). Similarly, when survival analysis was re-conducted, NLR and BLR were significantly associated with OS and PFS (Table S3).

**Table S1:** Receiver operating characteristic analysis of hematological markers for predicting survival

Variable	Cut-off	Specificity	Sensitivity	AUC
NLR*	3.4	80.9	77.8	83.1
ABCr*	0.035	80.9	88.9	83.3

Note:

* Among the 13 hematological markers used in this study, only NLR and ABCr produced receiver operating characteristic curves with optimal values that were significantly different from those obtained by chance.

Abbreviations: ABCr: Absolute basophil count ratio; AUC: Area under the curve; NLR: Neutrophil–to–lymphocyte ratio.

**Table S2:** Markers associated with treatment response (categorized according to receiver operating characteristic values)

Variable	Value	CR	DP	*p*-value	*OR*
NLR	Below ROC	17 (70.8%)	2 (33.3%)	0.156	0.206 (0.030 – 1.393)
	Above ROC	7 (29.2%)	4 (66.7%)		
ABCr	Below ROC	17 (70.8%)	1 (16.7%)	0.026*	0.082 (0.008 – 0.838)
	Above ROC	7 (29.2%)	5 (83.3%)		

Note:

* indicates statistical significance (*p*≤0.05).

Abbreviations: ABCr: Absolute basophil count ratio; CR: Complete response; DP: Disease progression; NLR: Neutrophil–to–lymphocyte ratio; *OR*: Odds ratio;

ROC: Receiver operating characteristic.

**Table S3:** Mean survival among groups categorized according to receiver operating characteristic values

Variable	Values	OS				PFS			
	
		Mean	Lower	Upper	*p*-value	Mean	Lower	Upper	*p*-value
NLR	Below ROC	70.8	60.7	81.1	0.001*	58.4	43.5	73.3	0.027*
	Above ROC	20.5	13.9	27.0		18.2	10.8	25.5	
ABCr	Below ROC	74.0	65.3	82.8	0.000*	62.4	48.3	76.5	0.015*
	Above ROC	26.1	12.6	39.6		24.8	9.1	40.6	

Note:

* indicates statistical significance (*p*≤0.05).

Abbreviations: ABCr: Absolute basophil count ratio; NLR: Neutrophil–to–lymphocyte ratio; *OR*: Odds ratio; OS: Overall survival; PFS: Progression-free survival; ROC: Receiver operating characteristic.

## 4. Discussion

To the best of the authors’ knowledge, this study was the first to report on the predictive value of hematological biomarkers in relation to survival and tumor response following definitive chemoradiation in vulvar carcinoma. Our results demonstrated that treatment responders had lower NLR values, while patients with disease recurrence exhibited higher ELR and BLR values compared to those without recurrence. ROC analysis revealed that both NLR and BLR had significant predictive power in distinguishing survival, with optimal cutoff values being 3.4 and 0.035, respectively. Within these cutoffs, both NLR and BLR were significantly associated with OS and PFS.

The dynamic link between inflammation and cancer is well established and has been recognized since the 19^th^ century when leukocytes were first observed within neoplastic tissue.^9^,^10^ Research suggests that the systemic inflammatory response plays a crucial role in all stages of neoplastic development and significantly influences the tumor microenvironment.^8^ Moreover, it appears that the interaction between neoplastic cells and their microenvironment is mediated by various immune cells (*e.g*., neutrophils, eosinophils, basophils, mast cells, monocytes, macrophages, dendritic cells, natural killer cells, and lymphocytes), highlighting the contradictory role of the immune system in both protecting against and promoting cancer progression.^11^

The status of the inflammatory response can be represented by various biomarkers, among which the NLR is particularly noteworthy. Composed of neutrophils (a proinflammatory marker that promotes tumorigenesis) and lymphocytes (which mediate inflammation and inhibit tumor proliferation), the NLR reflects the balance between the inflammatory and immune systems and may serve as a valuable prognostic tool.^12^ In this study, we demonstrated that NLR was predictive of both OS and PFS. Moreover, NLR values were significantly higher in patients who did not respond to treatment. These findings align with existing literature, which consistently shows that NLR is a prognostic factor of OS in both gynecological (*e.g*., ovarian and cervical) and non-gynecological cancers (*e.g*., breast, lung, and pancreatic).^13^ Other studies have also highlighted the predictive value of NLR for metastasis, cancer stage, and lymph node involvement.^8^ The role of neutrophils in promoting cancer cell invasion, migration, and angiogenesis, coupled with the impact of lymphopenia on neoplastic progression,^14^ may help explain the predictive power of NLR.^15^

In contrast to the consistent findings associated with NLR, the behavior of PLR in both non-gynecological and gynecological cancers shows significant variability. Thrombocytosis is thought to play a role in neoplastic progression and metastasis, particularly in ovarian cancer.^16^ Interestingly, tumor cells can initiate and enhance thrombopoiesis through the production of cytokines and platelet factors 4, indicating a bidirectional relationship between the tumors and platelets.^17^ The literature suggests that PLR, when combined with NLR, can help predict patient survival following definitive chemoradiation in cervical cancer.^18^ A recent meta-analysis by Zhang *et al*.^19^ demonstrated that PLR is a predictor of both OS and PFS based on pooled data from 10 studies. However, in the context of vulvar cancer, the prognostic value of PLR remains inconclusive. Some studies have shown that PLR is a significant predictor of lymph node involvement,^13^ while others found no association between PLR/thrombocytosis and outcomes in various cohorts of vulvar cancer patients.^15^,^16^ In addition, PLR has been shown to lack prognostic significance in cervical cancer.^20^ Our results are consistent with the latter observations, as PLR was not predictive of survival, treatment response, or metastasis in our study.

Among our participants, BLR was a significant predictor of survival, PFS, and response to treatment. This finding is coincident with several studies that have shown that higher basophil counts were associated with tumor stage, recurrence, lymph node involvement, and survival in malignancies, such as cervical, bladder, pancreatic, and prostate cancer.^21^-^24^ Winarto *et al*.^15^ also demonstrated that BLR was a significant predictor of tumor stage and lymph node metastasis in univariate models and distant metastasis in multivariate models among patients with vulvar cancer. Given the role of basophils and infiltrating lymphocytes in chronic inflammation, BLR might play a potential prognostic part in cancer.^25^ Therefore, a high BLR ratio may indicate an inadequate immune response to carcinogenesis. On the other hand, Rosner *et al*.^26^ reported that melanoma patients with higher basophil counts had better survival outcomes. Similarly, a low baseline basophil count has been associated with poorer survival outcomes and more aggressive disease in patients with non-metastatic colorectal cancer.^27^ Conversely, Li *et al*.^20^ found no prognostic value for BLR in patients with stage IIB cervical cancer.

Overall, the literature regarding the prognostic value of hematological markers in vulvar cancer is limited. Ertas *et al*.^13^ included a total of 64 patients with vulvar cancer from two different hospital databases. Of the participants, 64% were classified as FIGO stage I. The study found that NLR and PLR were significant predictors of lymph node metastasis. In addition, Winarto *et al*.^15^ examined the prognostic significance of 14 hematological markers on clinical staging, lymph node metastasis, and distant metastasis in 86 patients with vulvar cancer. They found that NLR was predictive of lymph node metastasis, whereas BLR was predictive of distant metastasis. Unlike our study, however, none of these studies included survival parameters. In addition to the differences in outcome variables, the limited predictive power of hematological markers in the literature on vulvar cancer can be largely attributed to small sample sizes.

The advanced stage of vulvar cancer is associated with high morbidity and significantly affects psychosexual health.^28^ Despite the accuracy of surgical staging, the decision to perform superficial and/or deep inguinal lymphadenectomy remains contested, which has led to the adoption of more conservative surgical approaches. Therefore, the ability to predict stages, distant metastasis, lymph node involvement, or survival, ideally using minimalistic and consistent measures, is of paramount importance. Pre-operative detection results of markers of the systemic inflammatory response, such as NLR, PLR, and BLR, are easily reproducible, fast, and cost-effective.^13^ These markers, among others, have demonstrated prognostic potential across various tumor types in the literature. Nonetheless, the literature on gynecological cancers in general, and vulvar cancer in particular, is highly heterogeneous. Cutoff values are determined using different methodologies (*e.g*., mean, median, ROC, and X-tile analysis) and are based on a range of outcome measures (*e.g*., mortality, distant metastasis, and lymph node involvement). Thus, before any of these markers can be used for prediction or risk stratification, rigorous validation of their cutoff values must be conducted using large-scale, preferably multicentric, cohorts.

On a separate note, locally advanced vulvar cancer has been associated with lower survival rates due to older mean age at diagnosis and a higher risk of microscopic distant metastases.^29^ Most patients present with a large or centrally-located primary tumor and bulky lymph nodes, which make radical surgery extremely challenging, with a high risk of residual disease and functional deficits. Definitive chemoradiation offers a reasonable radical treatment approach for this group of patients. Our cohort demonstrated 1-year OS and PFS rates similar to those reported in comparable literature.^30^ However, survival, with or without disease, significantly declines at the 3-year mark but remains stable until the 5-year mark.

The observed variance may be attributed to differences in systemic health-care capabilities or inherent clinical and socioeconomic characteristics of the cohorts. For example, the response to radiation therapy is known to vary between patients. One study has shown that a primary tumor volume exceeding 30 cc required replanning during the radiation course due to tumor shrinkage.^31^ This raises two important questions: first, should specific groups of vulvar cancer patients be analyzed separately due to their unique responses to treatment? Second, can hematological markers be used to predict the need for replanning and even determine the appropriate radiation dose? Future studies should aim to address these questions.

Finally, overexpression of cyclin-dependent kinase inhibitor 2A (p16) and HPV status have been shown to impact survival and response to radiation therapy in vulvar cancer patients.^32^,^33^ Only 6 patients (20%) were p16-positive.

Our findings should be interpreted with caution due to several limitations. First, the study had a relatively small sample size of patients, all of whom were recruited from a single institution and in a retrospective manner. The latter is particularly important because the authors cannot be fully confident in the exact timing or methodology used to collect pre-treatment hematological markers. Second, other inflammatory markers and variables, such as HPV status and CRP levels, were not considered. Third, the interactions between different hematological markers, particularly those based on lymphocyte ratios, were not assessed. Nonetheless, this was the first study to explore the potential predictive value of hematological markers in a homogeneously treated group of patients with locally advanced vulvar carcinoma who underwent definitive chemoradiation. Our findings highlight the need for larger, prospective studies to better evaluate these markers.

## 5. Conclusion

This study demonstrated the potential utility of pre-treatment hematological markers, such as NLR and BLR, as predictive tools for clinical and radiological responses to definitive chemoradiation in locally advanced vulvar cancer. Future prospective studies incorporating dose escalation for poor responders based on pre-treatment NLR are warranted.

## Data Availability

The data that support the findings of this study are available from the corresponding author, upon reasonable request.
